# Brain clocks capture diversity and disparity in aging and dementia

**DOI:** 10.21203/rs.3.rs-4150225/v1

**Published:** 2024-06-25

**Authors:** Sebastian Moguilner, Sandra Baez, Hernan Hernandez, Joaquín Migeot, Agustina Legaz, Raul Gonzalez-Gomez, Francesca R. Farina, Pavel Prado, Jhosmary Cuadros, Enzo Tagliazucchi, Florencia Altschuler, Marcelo Adrián Maito, María E. Godoy, Josephine Cruzat, Pedro A. Valdes-Sosa, Francisco Lopera, John Fredy Ochoa-Gómez, Alfredis Gonzalez Hernandez, Jasmin Bonilla-Santos, Rodrigo A. Gonzalez-Montealegre, Renato Anghinah, Luís E. d’Almeida Manfrinati, Sol Fittipaldi, Vicente Medel, Daniela Olivares, Görsev G. Yener, Javier Escudero, Claudio Babiloni, Robert Whelan, Bahar Güntekin, Harun Yırıkoğulları, Hernando Santamaria-Garcia, Alberto Fernández Lucas, David Huepe, Gaetano Di Caterina, Marcio Soto-Añari, Agustina Birba, Agustin Sainz-Ballesteros, Carlos Coronel-Oliveros, Amanuel Yigezu, Eduar Herrera, Daniel Abasolo, Kerry Kilborn, Nicolás Rubido, Ruaridh A. Clark, Ruben Herzog, Deniz Yerlikaya, Kun Hu, Mario A. Parra, Pablo Reyes, Adolfo M. García, Diana L. Matallana, José Alberto Avila-Funes, Andrea Slachevsky, María I. Behrens, Nilton Custodio, Juan F. Cardona, Pablo Barttfeld, Ignacio L. Brusco, Martín A. Bruno, Ana L. Sosa Ortiz, Stefanie D. Pina-Escudero, Leonel T. Takada, Elisa Resende, Katherine L. Possin, Maira Okada de Oliveira, Alejandro Lopez-Valdes, Brain Lawlor, Ian H. Robertson, Kenneth S. Kosik, Claudia Duran-Aniotz, Victor Valcour, Jennifer S. Yokoyama, Bruce L. Miller, Agustin Ibanez

**Affiliations:** 1 Latin American Brain Health Institute, Universidad Adolfo Ibañez, Santiago de Chile, Chile; 2 Cognitive Neuroscience Center, Universidad de San Andrés, Buenos Aires, Argentina; 3 Department of Neurology, Massachusetts General Hospital and Harvard Medical School, Boston, MA, USA; 4 Facultad de Odontología y Ciencias de la Rehabilitación, Universidad San Sebastián, Santiago de Chile, Chile; 5 Universidad de los Andes, Bogota, Colombia; 6 Global Brain Health Institute (GBHI), University of California, San Francisco, US; and Trinity College Dublin, Dublin, Ireland; 7 Grupo de Bioingeniería, Decanato de Investigación, Universidad Nacional Experimental del Táchira, San Cristóbal 5001, Venezuela; 8 Pontificia Universidad Javeriana (PhD Program in Neuroscience) Bogotá, San Ignacio, Colombia; 9 Center of Memory and Cognition Intellectus, Hospital Universitario San Ignacio Bogotá, San Ignacio, Colombia; 10 University of Buenos Aires, Argentina; 11 The Clinical Hospital of Chengdu Brain Sciences, University of Electronic Sciences; 12 Technology of China, Chengdu, China; Cuban Neuroscience Center, La Habana, Cuba; 13 Grupo de Neurociencias de Antioquia (GNA) University of Antioquia, Medellín, Colombia; 14 Department of Psychology, Master program of Clinical Neuropsychology, Universidad Surcolombiana Neiva, Neiva - Huila, Colombia; 15 Department of Psychology, Universidad Cooperativa de Colombia; 16 Neurocognition and Psychophysiology Laboratory, Universidad Surcolombiana, Neiva - Huila, Colombia; 17 Reference Center of Behavioural Disturbances and Dementia, School of Medicine, University of Sao Paulo, Sao Paulo, Brazil; 18 Traumatic Brain Injury Cognitive Rehabilitation Out-Patient Center, University of Sao Paulo, Sao Paulo, Brazil; 19 Center for Social and Cognitive Neuroscience, School of Psychology, Universidad Adolfo Ibáñez, Santiago, Chile; 20 Neuropsychology and Clinical Neuroscience Laboratory (LANNEC), Physiopathology program-Institute of Biomedical Sciences (ICBM), Neuroscience and East Neuroscience Departments, Faculty of Medicine, University of Chile, Santiago, Chile.; 21 Centro de Neuropsicología Clínica (CNC), Santiago, Chile; 22 Faculty of Medicine, Izmir University of Economics, 35330, Izmir, Turkey; 23 Brain Dynamics Multidisciplinary Research Center, Dokuz Eylul University, Izmir, Turkey; 24 Izmir Biomedicine and Genome Center, Izmir, Turkey; 25 School of Engineering, Institute for Imaging, Data and Communications, University of Edinburgh, Scotland, UK; 26 Department of Physiology and Pharmacology “V. Erspamer”, Sapienza University of Rome, Rome, Italy; 27 Hospital San Raffaele Cassino, Cassino, (FR), Italy; 28 School of Psychology, Trinity College Dublin, Dublin 2, Ireland; 29 Department of Neurosciences, Health Sciences Institute, Istanbul Medipol University, İstanbul, Turkey; 30 Health Sciences and Technology Research Institute (SABITA), Istanbul Medipol University, Istanbul, Turkey; 31 Department of Biophysics, School of Medicine, Istanbul Medipol University; 32 Departamento de Medicina Legal, Psiquiatría y Patología, Facultad de Medicina, Universidad Complutense de Madrid; 33 Center for Social and Cognitive Neuroscience (CSCN), School of Psychology, Universidad Adolfo Ibáñez; 34 Department of Electronic and Electrical Engineering, University of Strathclyde, Glasgow, UK; 35 Universidad Católica San Pablo, Arequipa, Peru; 36 Centro Interdisciplinario de Neurociencia de Valparaíso (CINV), Universidad de Valparaíso, Chile.; 37 Trinity College Dublin, The University of Dublin, Dublin, Ireland; 38 Departamento de Estudios Psicológicos, Universidad ICESI, Cali, Colombia; 39 Centre for Biomedical Engineering, School of Mechanical Engineering Sciences, Faculty of Engineering and Physical Sciences, University of Surrey, Guildford GU2 7XH, UK; 40 School of Psychology, University of Glasgow, Glasgow, Scotland; 41 Institute for Complex Systems and Mathematical Biology, University of Aberdeen, Aberdeen, AB24 3UE, UK; 42 Centre for Signal and Image Processing, Department of Electronic and Electrical Engineering, University of Strathclyde, UK; 43 Sorbonne Université, Institut du Cerveau - Paris Brain Institute - ICM, Inserm, CNRS, Paris, France; 44 Department of Neurosciences, Health Sciences Institute, Dokuz Eylül University, Izmir, Turkey; 45 Harvard Medical School, Boston, USA; 46 Department of Psychological Sciences and Health, University of Strathclyde, Glasgow, United Kingdom; Researcher associate of BrainLat, Universidad Adolfo Ibáñez, Santiago, Chile; 47 Departamento de Lingüística y Literatura, Facultad de Humanidades, Universidad de Santiago de Chile, Santiago, Chile 2; 48 Department of Geriatrics. Instituto Nacional de Ciencias Médicas y Nutrición Salvador Zubirán. Mexico City, Mexico.; 49 Memory and Neuropsychiatric Center (CMYN), Neurology Department, Hospital del Salvador & Faculty of Medicine, University of Chile, Santiago, Chile; 50 Geroscience Center for Brain Health and Metabolism (GERO), Santiago, Chile; 51 Neuropsychology and Clinical Neuroscience Laboratory (LANNEC), Physiopathology Program – Institute of Biomedical Sciences (ICBM), Neuroscience and East Neuroscience Departments, Faculty of Medicine, University of Chile, Santiago, Chile; 52 Neurology and Psychiatry Department, Clínica Alemana-Universidad Desarrollo, Santiago, Chile; 53 Centro de Investigación Clínica Avanzada (CICA), Facultad de Medicina-Hospital Clínico, Universidad de Chile, Independencia, Santiago, 8380453, Chile; 54 Departamento de Neurología y Neurocirugía, Hospital Clínico Universidad de Chile, Independencia, Santiago, 8380430, Chile; 55 Departamento de Neurociencia, Facultad de Medicina, Universidad de Chile, Independencia, Santiago, 8380453, Chile; 56 Servicio de Neurología, Instituto Peruano de Neurociencias, Lima, Perú.; 57 Facultad de Psicología, Universidad del Valle, Santiago de Cali, Colombia; 58 Cognitive Science Group. Instituto de Investigaciones Psicológicas (IIPsi), CONICET UNC, Facultad de Psicología, Universidad Nacional de Córdoba, Boulevard de la Reforma esquina Enfermera Gordillo, CP 5000. Córdoba, Argentina; 59 Centro de Neuropsiquiatría y Neurología de la Conducta (CENECON), Facultad de Medicina, Universidad de Buenos Aires (UBA), C.A.B.A., Buenos Aires, Argentina.; 60 Instituto de Ciencias Biomédicas (ICBM) Facultad de Ciencias Médicas, Universidad Catoóica de Cuyo, San Juan, Argentina; 61 Instituto Nacional de Neurologia y Neurocirugia MVS, Universidad Nacional Autonoma de Mexico, Mexico, Mexico.; 62 Memory and Aging Center, Department of Neurology, Weill Institute for Neurosciences, University of California, San Francisco, California, USA; 63 Cognitive and Behavioral Neurology Unit, Hospital das Clinicas, University of São Paulo Medical School, São Paulo, Brazil; 64 Universidade Federal de Minas Gerais, Belo Horizonte, Minas Gerais, Brazil; 65 The University of California Santa Barbara (UCSB), California, USA; 66 The University of Chicago, Division of the Biological Sciences, 5841 S Maryland Avenue Chicago, IL 60637, USA

## Abstract

Brain clocks, which quantify discrepancies between brain age and chronological age, hold promise for understanding brain health and disease. However, the impact of multimodal diversity (geographical, socioeconomic, sociodemographic, sex, neurodegeneration) on the brain age gap (BAG) is unknown. Here, we analyzed datasets from 5,306 participants across 15 countries (7 Latin American countries -LAC, 8 non-LAC). Based on higher-order interactions in brain signals, we developed a BAG deep learning architecture for functional magnetic resonance imaging (fMRI=2,953) and electroencephalography (EEG=2,353). The datasets comprised healthy controls, and individuals with mild cognitive impairment, Alzheimer’s disease, and behavioral variant frontotemporal dementia. LAC models evidenced older brain ages (fMRI: MDE=5.60, RMSE=11.91; EEG: MDE=5.34, RMSE=9.82) compared to non-LAC, associated with frontoposterior networks. Structural socioeconomic inequality and other disparity-related factors (pollution, health disparities) were influential predictors of increased brain age gaps, especially in LAC (R^2^=0.37, F^2^=0.59, RMSE=6.9). A gradient of increasing BAG from controls to mild cognitive impairment to Alzheimer’s disease was found. In LAC, we observed larger BAGs in females in control and Alzheimer’s disease groups compared to respective males. Results were not explained by variations in signal quality, demographics, or acquisition methods. Findings provide a quantitative framework capturing the multimodal diversity of accelerated brain aging.

The brain undergoes dynamic functional changes with age^1–3^. Accurately mapping the trajectory of these changes and how they relate to chronological age is critical for understanding the aging process, multilevel disparities^4,5^, and brain disorders^1^ such as the Alzheimer’s disease continuum, which includes mild cognitive impairment (MCI), and related disorders like behavioral variant frontotemporal dementia (bvFTD)^6^. Brain clocks or brain age models have emerged as dimensional, transdiagnostic metrics that measure brain health influenced by a range of factors^7–9^, suggesting that they may be able to capture multimodal diversity^10^. Notably, underrepresented populations from Latin American countries (LAC) exhibit higher genetic diversity and distinct physical, social and internal exposomes^11,12^ that impact brain phenotypes^4,13,14^. Income and socioeconomic inequality^15,16^, high levels of air pollution^17^, limited access to timely and effective healthcare^18^, increased prevalence of communicable diseases^19^, rising prevalence of non-communicable diseases^19,20^, and low education attaiment^21,22^, are determinants of brain health in LAC^18^. Thus, although measuring the brain age gap (BAG) could enhance our understanding of disease risk and its impact on accelerated aging^23^, there is a lack of research on brain age models in underrepresented populations with increased socioeconomic and health disparities^18,24,25^.

Sex and gender differences emerge as critical factors influencing brain changes. Studies on atrophy in Alzheimer’s disease continuum reveal a faster rate of brain atrophy in females than in males^26^. Moreover, country-level gender inequality is associated to sex differences in cortical thickness^27^. Structural gender inequality further impacts brain health, with adverse environments affecting dendritic branching and synapse formation^28^. However, no studies to date have explored the spectrum of brain age abnormalities, including the effects of demographic heterogeneity across geographical regions, sexes, and the continuum from brain health to disease. Further, most studies have been conducted with participants from the global north, resulting in a lack of generalization to underrepresented populations from LAC^24,29–31^.

Multimodal machine learning studies show promise in brain aging^23^; however, most rely on structural MRI, overlooking brain network dynamics. Complex spatiotemporal dimensions can be tracked with spatial accuracy through functional magnetic resonance imaging (fMRI) and millisecond precision using electroencephalogram (EEG)^32^. Given the complementary strengths of fMRI and EEG, it is crucial to cross-validate existing brain clock models using these techniques. However, no studies have simultaneously applied EEG and fMRI to replicate brain age effects. Additionally, standard machine learning approaches are less generalizable than deep learning methods^33^. Brain age indices has been restricted by the predominant use of MRI or PET, which are less accessible and affordable in LAC, leading to selection biases^34^. EEG offers a solution due to its cost-effectiveness, portability, and ease of implementation in aging and dementia^35,36^. However, few studies have combined accessible techniques with deep learning to develop scalable brain age markers. The application of EEG is hindered by heterogeneity in recordings, electrode layouts, acquisition systems, processing pipelines, and small sample sizes^37^. These standardization challenges have impeded the integration of fMRI and EEG in extensive, multicenter brain age research.

We adopted a framework to tackle diversity by including datasets from LAC and non-LAC regions (n = 5306), utilizing graph convolutional networks (GCN) to functional connectivity of fMRI and EEG signals. We hypothesized that, across fMRI and EEG imaging, models would accurately predict BAGs and be sensitive to the impacts of multimodal diversity, including geographical and sociodemographic effects, sex differences, health disparities, and exposome influences. By testing this hypothesis, we aimed to assess the effectiveness of high-order interactions and deep learning in predicting brain age differences across diverse and heterogeneous populations of healthy aging and neurocognitive disorders.

## Results

**Fig. 1.**
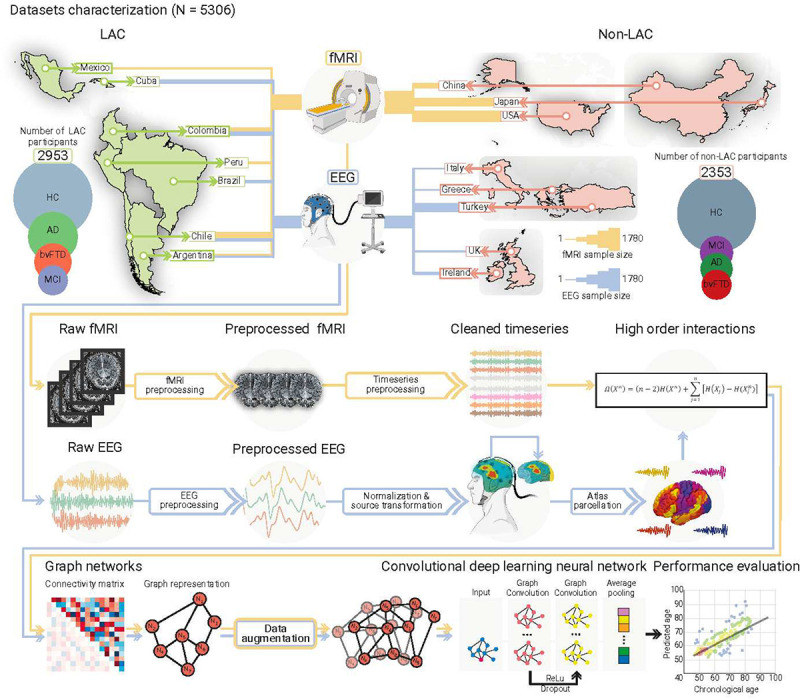
Datasets characterization and analysis pipeline. Datasets included Latin American countries (LAC) and non-LAC healthy controls (HC, total N = 3509) and participants with Alzheimer’s disease (AD, total N = 828), behavioral variant frontotemporal dementia (bvFTD, total N = 463), and mild cognitive impairment (MCI, total N = 517). The functional magnetic resonance imaging dataset (fMRI, yellow lines) included 2953 participants from LAC (Argentina, Chile, Colombia, Mexico, and Peru) as well as non-LAC (the USA, China, and Japan). The electroencephalography dataset (EEG, blue lines) involved 2353 participants from Argentina, Brazil, Chile, Colombia, and Cuba (LAC) as well as Greece, Ireland, Italy, Turkey, and the UK (non-LAC). Circles represent the number of participants per group, scaled between the number of participants in the largest and smallest groups for each region to facilitate visualization. Line thickness represents the number of participants with fMRI (yellow lines) and EEG (blue lines) per country. The raw fMRI and EEG signals were preprocessed by filtering and artifact removal and the EEG signals were normalized to project them into source space. A parcellation using the automated anatomical labeling (AAL) atlas for both the fMRI and EEG signals was performed to build the nodes from which we calculated the high-order interactions using the Ω-information metric. A connectivity matrix was obtained for both modalities, which was later represented by graphs. Data augmentation was performed only in the testing dataset. The graphs were used as input for a graph convolutional deep learning network (architecture shown in the last row), with separate models for EEG and fMRI. Finally, age prediction was obtained, and the performance was measured by comparing the predicted vs. the chronological ages. This figure was partially created using Biorender under Team license.

We employed resting-state fMRI (n = 2953) and EEG (n = 2353) signals separately to evaluate whether a deep-learning computational pipeline (Fig. 1) captures differences in brain aging across heterogeneous populations. We included fMRI data from 2953 participants from Argentina, Chile, Colombia, Mexico, and Peru (LAC) and the USA, China, and Japan (non-LAC). The EEG dataset involved 2353 participants from Argentina, Brazil, Chile, Colombia, and Cuba (LAC), and Greece, Ireland, Italy, Turkey, and the UK (non-LAC). Healthy controls, MCI, Alzheimer’s disease, and bvFTD groups were included. We focused on the Alzheimer’s disease and bvFTD as these conditions represent the most common late-onset and early-onset causes of dementia^38,39^. We included the Alzheimer’s disease continuum, which encompasses MCI, to capture the prodromal stages of the disease^39^. Raw fMRI and EEG signals were preprocessed to remove artifacts and then normalized. Based on multivariate information theory, we calculated high-order interactions^1^. Weighted graphs were used as inputs for a graph convolutional deep learning network trained to predict brain age, employing one model for fMRI and another for EEG.

### BAG across LAC and non-LAC datasets

**Fig. 2.**
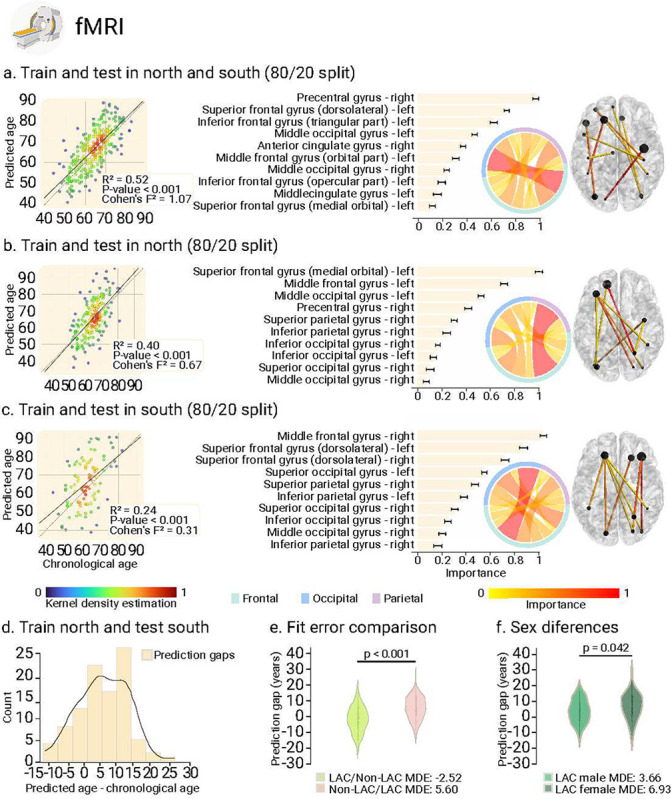
fMRI training and testing the deep learning model in different samples. **(a)** Ordinary least squares (OLS) regression comparing chronological age vs. predicted age with the feature importance list for training and testing in the whole sample. **(b)** Regression comparing chronological age vs. predicted age with the feature importance list for training and testing in the non-LAC dataset. **(c)** Regression comparing chronological age vs. predicted age with the feature importance list for training and testing in the LAC dataset. For **(a)**, **(b)** and **(c),** data point colors indicate the kernel density estimation to provide a visual representation of the density of prediction errors across different values of chronological age. The bars show the brain region feature importance list in descending order, with ring plots and glass brain representations of the most important network-edge connections. **(d)** Histogram of the prediction error when training in non-LAC dataset and testing in LAC dataset. **(e)** Violin plot of the distribution and statistical comparison of training and testing with different regions using a permutation test (5000 iterations). **(f)** Violin plot of the distribution and statistical comparison of testing the models on females and males using a permutation test (5000 iterations). LAC = Latin American countries.

**Fig. 3.**
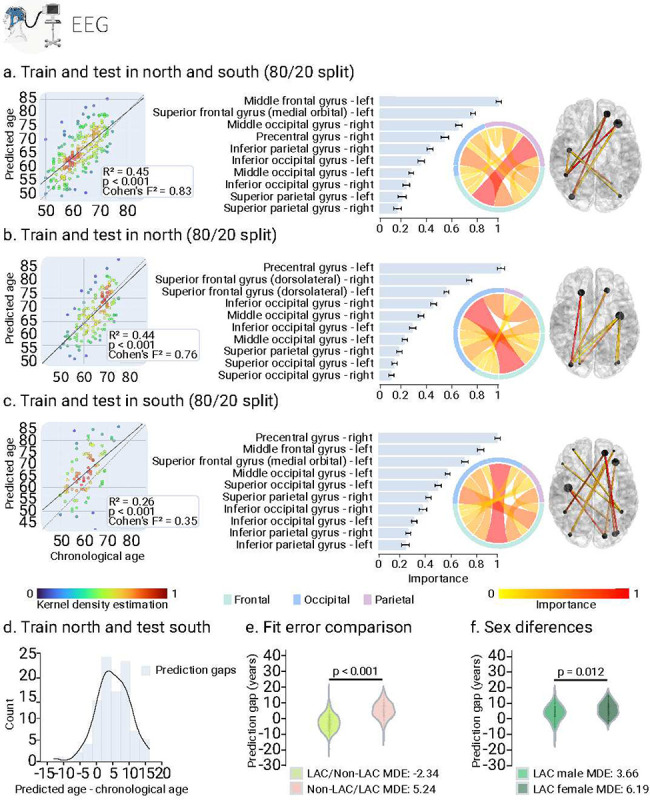
EEG training and testing the deep learning model in different samples. **(a)** Ordinary least squares (OLS) regression comparing chronological age vs. predicted age with the feature importance list for training and testing in the whole sample. **(b)** Regression comparing chronological age vs. predicted age with the feature importance list for training and testing in the non-LAC dataset. **(c)** Regression comparing chronological age vs. predicted age with the feature importance list for training and testing in the LAC dataset. For **(a)**, **(b)** and **(c),** data point colors indicate the kernel density estimation to provide a visual representation of the density of prediction errors across different values of chronological age. The bars show the brain region feature importance list in descending order, with ring plots and glass brain representations of the most important network-edge connections. **(d)** Histogram of the prediction error when training in non-LAC dataset and testing in LAC dataset. **(e)** Violin plot of the distribution and statistical comparison of training and testing with different regions using a permutation test (5000 iterations). **(f)** Violin plot of the distribution and statistical comparison of testing the models on females and males using a permutation test (5000 iterations). LAC = Latin American countries.

We used the fMRI and EEG signals from the control’s datasets (i.e., LAC and non-LAC) to train and test brain-aging models. We employed 80% cross-validation with a 20% hold-out testing split. As shown in Figs. 2a and 3a, our models predicting brain age obtained adequate goodness of fit (fMRI: R^2^ = 0.52, *p* < 0.001, F^2^ = 1.07; EEG: R^2^ = 0.45, *p* < 0.001, F^2^ = 0.83). We implemented the Root Mean Square Error (RMSE) to evaluate models’ fit, obtaining acceptable brain age predictions (fMRI-RMSE = 7.24, EEG-RMSE = 6.45). For both, fMRI and EEG, the main predictive brain-regional features included hubs in frontoposterior networks (nodes in precentral gyrus, the middle occipital gyrus, and the superior and middle frontal gyri; Fig. 2a and 3a). Additional nodes for the fMRI model included the inferior frontal gyri, and the anterior and median cingulate and paracingulate gyri (Fig. 2a.). For EEG, key nodes also comprised the superior and inferior parietal gyri and the inferior occipital gyrus (Fig. 3a). Thus, for both fMRI and EEG the models showed an adequate fit and predictive performance, with key predictive features involving frontoposterior networks in the brain.

### BAG in non-LAC datasets

Using the same data split ratio, we trained and tested the models in non-LAC datasets. As shown in Figs. 2b and 3b, our models predicting brain age yielded considerable goodness of fit (fMRI: R^2^ = 0.40, *p* < 0.001, F^2^ = 0.67; EEG: R^2^ = 0.43, *p* < 0.001, F^2^ = 0.76). RMSE values were also adequate (fMRI-RMSE = 8.66; EEG-RMSE = 6.54). Mean Directional Errors (MDE) for fMRI and EEG were 0.69 and 1.07, respectively. For both fMRI and EEG, the main predictive features included hubs in frontoposterior networks including the superior frontal gyrus (dorsolateral), the precentral gyrus, and the middle occipital gyrus (Fig. 2b and 3b). Additional critical nodes for the fMRI model included the inferior and middle frontal gyri, and the anterior and median cingulate and paracingulate gyri (Fig. 2b). For EEG, key nodes also comprised the superior and inferior occipital gyri, and the superior parietal gyrus (Fig. 3b). In brief, models trained on non-LAC datasets exhibited strong fit values and predictive features as in the overall dataset analysis.

### BAG in LAC datasets

When trained and tested in the LAC datasets (Figs. 2c and 3c), models demonstrated moderate goodness of fit indexes but were less precise, as indicated by higher RMSE values (fMRI = 11.91; EEG = 9.82). We observed increased positive biases in the MDE measures compared to the non-LAC models (fMRI = 3.18; EEG = 5.34). Again, the main features involved frontoposterior networks. Common nodes for fMRI and EEG included the superior and middle occipital gyri, the superior and inferior parietal gyri, and the superior and middle frontal gyri (Fig. 2c and 3c). For EEG, the model also highlighted the precentral gyrus, and the inferior occipital gyrus (Fig. 3c). Thus, models trained on LAC datasets showed moderate fit and positive biases (older brain age) in frontotemporal nodes (fMRI and EEG), compared to non-LAC models.

### Cross-regional effects in model generalization

**Fig. 4.**
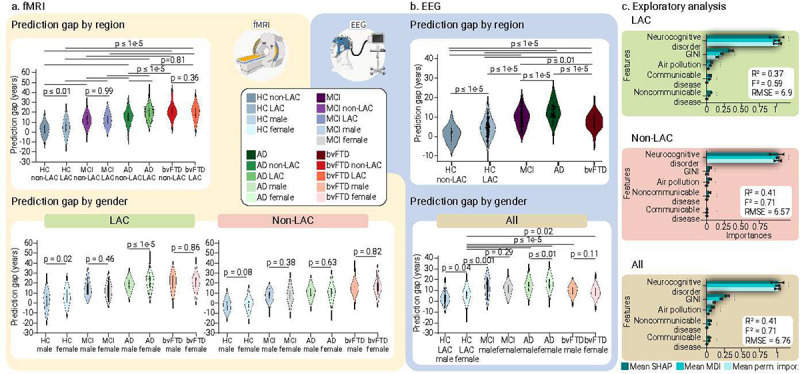
Groups, sex, and macrosocial influences in BAGs. Violin plots for the distribution of prediction gaps for different groups and sex effects using **(a)** fMRI and **(b)** EEG datasets. The statistical comparisons were calculated using subsample permutation testing with 5000 iterations. **(c)** Associations between macrosocial and disease disparity factors with BAGs were assessed with a multi-method approach comprising SHAP values, feature importance (mean decrease in impurity, MDI), and permutation importance. Plots show the mean importance values for each method, along with their 99% confidence interval, as well as the average R-squared and Cohen’s F^2^. * = Significant predictors. Shaded bars indicate significance across the three methods. LAC = Latin American countries, HC non-LAC = Healthy controls from non-LAC, HC LAC = Healthy controls from LAC, MCI = mild cognitive impairment, AD = Alzheimer’s disease, bvFTD = behavioral variant frontotemporal dementia, M = Males. F = Females, * p < 0.05, ** p < 0.01, *** p < 0.001.

We investigated the effects of cross-region training and testing with data from non-LAC and LAC. Training with non-LAC data and testing on LAC data led to biases predicting older brain ages than chronological ages as shown by positive MDE values (Figs. 2d and 3d; fMRI: MDE = 5.60, RMSE = 9.44; EEG: MDE = 5.24, RMSE = 7.23). On the contrary, training on LAC and testing on non-LAC resulted in negative age biases predicting younger brain age shown by the MDE (Figs. 2d and 3d; LAC/non-LAC fMRI: MDE = −2.52, RMSE = 8.41; LAC/non-LAC EEG: MDE = −2.34, RMSE = 5.69). Sex differences were observed in the BAG when training in the non-LAC and testing in LAC (Figs. 4a and 4b). Specifically, female participants in LAC exhibited a greater bias towards older brain age than males (fMRI: *p* = 0.04; EEG: *p* = 0.03). In conclusion, training with non-LAC data and testing on LAC data resulted in a bias towards predicting older brain ages, especially for female participants in LAC.

### Accelerated aging in MCI, Alzheimer’s disease and bvFTD

We investigated the effects of testing the controls-trained model (80%) on different subsamples, matched by age, sex, and education, from other groups (i.e., controls non-LAC, controls LAC, MCI, Alzheimer’s disease, and bvFTD, Table 1). Permutation subsample analyses with 5000 iterations revealed statistically significant BAGs between the non-LAC and LAC control groups (Figs. 4a and 4b, fMRI: *p* < 0.01; EEG: *p* < 1e-5). This difference was also observed for Alzheimer’s disease in the fMRI dataset (*p* < 1e-5). Additionally, for fMRI, we found significant differences between controls from non-LAC and all clinical groups from the same region [MCI (*p* < 1e-5), Alzheimer’s disease (*p* < 1e-5), and bvFTD (*p* < 1e-5)]. Similarly, for both fMRI and EEG, we observed significant differences between controls from LAC and all the clinical groups [fMRI: MCI (*p* < 1e-5), Alzheimer’s disease (*p* < 1e-5), and bvFTD (*p* < 1e-5); EEG: MCI (*p* < 1e-5), Alzheimer’s disease (*p* < 1e-5), and bvFTD (*p* < 0.01)]. Across fMRI and EEG datasets, both LAC and non-LAC, we observed a gradient of increasing brain age from controls to MCI to Alzheimer’s disease. The MCI groups significantly differed from Alzheimer’s disease (fMRI and EEG: *p* < 1e-5) and bvFTD (fMRI: *p* < 1e-5; EEG: *p* < 0.01), with older brain ages for Alzheimer’s disease and bvFTD. For the fMRI and EEG non-LAC datasets, the Alzheimer’s disease group also showed an older brain age than the bvFTD group (*p* < 0.01). Thus, larger brain age gaps were observed in LAC compared to non- LAC groups and across clinical groups, with a gradient of increasing brain age from controls to MCI to dementia.

### Sex differences in neurocognitive disorders

For fMRI, we analyzed the differences between male and female participants with the same diagnosis for the non-LAC and LAC datasets. There were no significant differences among groups from non-LAC datasets (Figs 4a and 4b). However, Alzheimer’s disease females from LAC exhibited significantly greater BAGs compared to males (fMRI: *p* < 1e-3, EEG: *p* < 0.001). No other significant effects were observed. We conducted a supplementary analysis incorporating country-level gender inequality (GII indexes), sex, region (LAC vs. non-LAC), and individual neurocognitive status (HC vs. MCI, Alzheimer’s disease, or bvFTD) as predictors of BAGs. The model demonstrated good performance (R^2^ = 0.40, F^2^ = 0.66, RMSE = 6.85, *p* < 1e-15) and all predictors were influential. Having a neurocognitive disorder and being a female living in countries with high gender inequality – particularly from LAC – were associated with higher BAGs (Extended Data Fig.1 and Supplementary Table 1). Overall, females with Alzheimer’s disease from LAC exhibited significantly greater brain age gaps compared to males, influenced by high gender inequality in their countries.

### Exposome determinants of BAGs

We employed gradient boosting regression models to explore the influence of physical and social exposomes, as well as disease disparity factors on BAGs. Predictors included aggregate country-level measures of air pollution (PM2.5), socioeconomic inequality (GINI index), and burdens of communicable, maternal, prenatal, and nutritional conditions, and non-communicable diseases. We also leveraged the individual neurocognitive status (HC versus Alzheimer’s disease, MCI, or bvFTD). We assessed predictors’ importance using a multi-method approach comprising permutation importance, mean decrease in impurity (MDI), and SHAP values (Fig. 4c). Across both LAC and non-LAC datasets, the models (R^2^ = 0.41, F^2^ = 0.71, RMSE = 6.76, F = 304.25, *p* < 1e-15) identified neurocognitive disorders (MCI, Alzheimer’s disease, or bvFTD) and higher socioeconomic inequality (GINI index) as the most influential and consistent predictors of increased BAGs (Fig. 4c). High levels of pollution and burden of non-communicable and communicable diseases were also predictive of increased BAGs, albeit less impactful. Stratified models for LAC (R^2^ = 0.37, F^2^ = 0.59, RMSE = 6.9, F = 138.78, *p* < 1e-15) and non-LAC (R^2^ = 0.41, F^2^ = 0.71, RMSE = 6.57, F = 135.91, *p* < 1e-15) also showed good performance, with neurocognitive disorders being the most influential predictor in both. In LAC, higher socioeconomic inequality was the second most consistent and influential predictor of larger BAGs across the three models. Air pollution and burden of communicable and non-communicable diseases were also influential. None of these variables was influential predictors in the non-LAC models. Predictors’ estimation coefficients are presented in Supplementary Table 2. In sum, neurocognitive disorders, followed by macrosocial factors linked to socioeconomic inequality, air pollution, and health disparities, were influential predictors of increased brain age gaps, especially in LAC.

### Sensitivity analyses

**Fig. 5.**
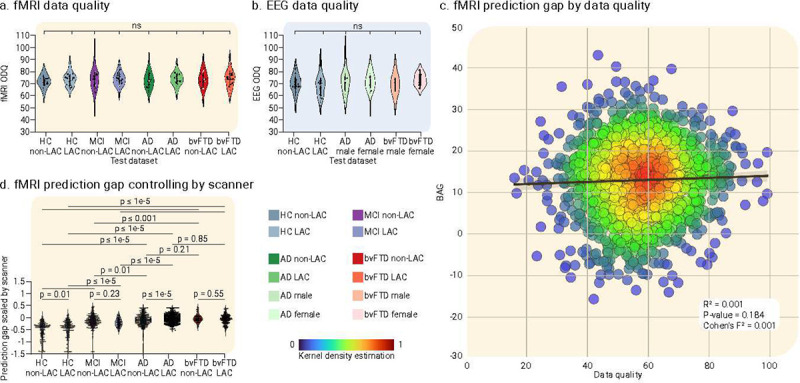
Sensitivity analysis. Violin plots for the distribution of data quality metrics of **(a)** fMRI and **(b)** EEG datasets. Both panels indicate null results between groups in terms of data quality. **(c)** Linear regression effects of scanner type, evidencing that the fMRI data quality was not significantly associated with fMRI BAGs differences. **(d)** fMRI BAG differences across groups controlling for scanner differences. The statistical comparisons were calculated using subsample permutation testing with 5000 iterations. LAC = Latin American countries, HC = Healthy controls, MCI = mild cognitive impairment, AD = Alzheimer’s disease, bvFTD = behavioral variant frontotemporal dementia.

We performed multiple tests to assess the validity of the results. First, we investigate whether variations in fMRI or EEG data quality explained the differences in brain age between the non-LAC and LAC. Subsample permutation tests with 5000 iterations showed no significant differences between any of the groups for fMRI (Fig. 5a) or EEG (Fig. 5b) data quality metrics. In addition, a linear regression examining scanner type effects showed that the fMRI data quality metric did not predict the BAGs (R^2^ = 0.001, *p* = 0.18, Cohen’s F^2^ = 0.001, Fig. 5c). To further test for scanner effects, we implemented a harmonization strategy by normalizing the BAG variable within each scanner type. We used the min-max scaler to ensure consistent minimum and maximum values across scanners. Results using this harmonization (Fig. 5d) and our initial approach were very similar. Additional analyses controlling for datasets collected with eyes open versus eyes closed protocols revealed no significant differences in BAGs across any groups (Extended Data Fig. 2).

We also controlled for effects of age and years of education on fMRI and EEG BAGs by including them as covariates in the group comparisons. All reported group differences remained significant after covariate adjustment (Supplementary Table 3). Years of education did not change the results for any analyses. In eight of the nine analyses, age did not have a significant effect. Considering the chronological age differences between Alzheimer’s disease and MCI groups, we performed a sensitivity analysis using a subset of MCI participants (fMRI: n = 254, mean age = 73.287 +/− 7.517; EEG: n = 52, mean age = 63.231 +/− 6.549) age matched to Alzheimer’s disease participants (fMRI: n = 254, mean age = 72.295 +/− 7.530, *p* = 0.13; EEG: n = 52, mean age = 62.769 +/− 6.302, *p* = 0.71). These results (Extended Data Fig. 3) confirmed those reported for the overall MCI and Alzheimer’s disease datasets (Figs. 4a and 4b). For both fMRI and EEG datasets, we found significantly larger BAGs in Alzheimer’s disease compared to MCI (fMRI: *p* < 1e-5; EEG: *p* < 0.01). For fMRI, these differences were observed in both LAC (*p* < 1e-5) and non-LAC (*p* < 1e-5) datasets. We also found differences between MCI participants from LAC vs. non-LAC (*p* < 1e-5) and Alzheimer’s disease participants from LAC vs. non-LAC (*p* < 1e-5). Thus, controlling for data quality, scanner effects, age, and education confirmed that the reported effects in brain age gaps remained the same.

## Discussion

Our study used brain clocks to capture diversity and disparity across LAC and non-LAC datasets using fMRI and source-space EEG techniques. Despite heterogeneity in signal acquisition and methods, we captured patterns of brain age modulations in healthy aging from diverse datasets and participants with MCI, Alzheimer’s disease, and bvFTD. Models trained and tested on non-LAC data showed greater convergence with chronological age. Conversely, models applied to LAC datasets indicated larger BAGs, suggesting accelerated aging. We observed a gradient of BAGs from controls to MCI to Alzheimer’s disease. Sex differences revealed an increased BAG in females in control and Alzheimer’s disease groups. Most brain clock patterns were independently confirmed and replicated across fMRI and EEG. Aggregate-level macrosocial factors, including socioeconomic inequality, pollution, and burden of communicable/non-communicable conditions modulated the BAG, especially in LAC. Variations in signal quality, demographics, or acquisition methods did not account for the results. The findings offer a framework that captures the multimodal diversity associated with accelerated aging in various global settings.

Our results suggest that being from LAC is associated with accelerated aging. The better fit of the non-LAC compared to the LAC models supports the notion that universal models of brain phenotypes do not generalize well to underrepresented populations^24,29,40^. Diversity-related factors associated with different exposome and disease disparities^4,10,24,41^ may influence the BAGs in LAC and non-LAC. Neurocognitive disorders played a crucial role^4,42^. However, structural socioeconomic inequality, a distinctive characteristic of LAC^15^, increased levels air pollution^43^, and the burden of non-communicable^19,20^ and communicable^18,44^ diseases also have an significant impact on BAGs. The fact that these effects were larger in LAC suggests that underlying inequalities and adverse environmental and health conditions play a macrosocial, structural driving role^11^ in the observed regional differences. Immigration may also influence brain age through social determinants of health^45^ and genetic diversity. In LAC, tricontinental admixtures lead to significant ancestral diversity within and across countries^46^, impacting dementia prevalence and brain phenotypes^41^. Future studies should consider these potential effects in BAGs.

Selective brain networks were associated with larger BAG in the clinical groups. Both fMRI and EEG models of BAGs yielded large-scale frontoposterior high-order interactions^1^, consistent with models of brain age involving long-range connections between frontal, cingular, parietal, and occipital hubs, which may be more vulnerable to aging effects^47–49^. Also consistent with the cumulative nature of neurobiological changes over time^50^, BAGs increased from controls through MCI to Alzheimer’s disease. A previous deep learning study using MRI and PET in participants with MCI and dementia also indicated increased brain age associated with disease progression^23^. Our results point to the brain age of MCI as being an intermediate stage between healthy aging and dementia^39^, and suggest that both fMRI and EEG markers of brain age may help identify groups at greater risk of progressing to dementia.

Sex and gender have been linked to poorer brain health outcomes^27,51^. Larger BAGs in controls and Alzheimer’s disease females from LAC may relate to sex-specific conditions such as menopause, which involves brain volume reduction and increased amyloid-beta deposition^52,53^. Females also exhibit disproportionate tau brain burden^54^, pronounced inflammatory dysregulation^55^ and lower basal autophagy^56^, all of which increase Alzheimer’s disease risk. Such sex-specific factors are intertwined with environmental factors and gender inequalities^51^. Females in countries with higher gender inequality exhibit greater cortical atrophy^27^. Our sex effects were specific for Alzheimer’s disease and LAC, consistent with the impacts of environmental^41^ versus genetic risks^57^ in Alzheimer’s disease and bvFTD, respectively. Despite advances in gender equality, women in LAC still face significant obstacles^58^ including lower education, less income and healthcare access, and greater caregiving burden, potentially exacerbating brain health issues and Alzheimer’s disease risk^59,60^. Previous models for brain age have been conducted predominantly in high-income settings, ignoring sex and gender differences triggered by region-specific influences^30,31^. Thus, the inclusion of diverse samples can help to better understand the biological and environmental interaction of sex and gender disparities.

Our study had different strengths. We used diverse datasets across LAC and non-LAC including 15 countries, featuring large sample sizes, and replicated results across fMRI and EEG. Geographical and sex differences modulated brain clocks across fMRI and EEG models, with more accelerated aging observed in controls and Alzheimer’s disease females from LAC, contributing to the understanding of the effects of sex and diversity in aging. We used an integrative approach to analyze fMRI and EEG data across a large and geographically diverse sample. The convergence of two neuroimaging techniques and population heterogeneity enhanced the generalizability of our findings, making a significant contribution to computational models that capture diversity^10^. Brain clocks based on high-order interactions capture many risks to brain health, and thus, offer a new approach to personalized medicine, particularly for underrepresented populations. Our framework combines multiple dimensions of diversity in brain health, the Alzheimer’s disease continuum and related disorders within a single measure of brain clocks, which is relevant for global health policies, generalizable computational models, and public health strategies. Incorporating EEG offers affordable and scalable solutions for disadvantaged settings, such as those in LAC, compared to traditional neuroimaging techniques^1,35^. Accessible metrics of accelerated aging can offer personalized assessments of diversity, aging, and neurocognitive disorders.

This study has multiple limitations. Our EEG dataset lacks representation from clinical groups in non-LAC, which may limit the generalizability. This issue is partially mitigated by the consistent results from the fMRI data, which included MCI, Alzheimer’s disease, and bvFTD groups from both regions. Our BAG approach is unimodal. Future research should adopt multimodal approaches to deepen our understanding of brain aging across different pathophysiological mechanisms^1^. We leveraged two independent training and test datasets with fMRI and EEG, with out-of-sample validation yielding consistent results across geographical comparisons, sex effects, and clinical conditions. These datasets involve multimodal settings and recording parameters, suggesting that our results are strong across highly variable conditions. However, future research should include more regions to further validate and strengthen our findings. Additionally, we did not include individual-level data on gender identity, socioeconomic status, and ethnic stratification. Future research incorporating these variables could further enrich our understanding of brain age across diverse populations. Lastly, the sex differences observed between controls from LAC and non-LAC exhibited moderate effect sizes. Further research should assess sex differences in other regions.

In conclusion, brain clock models were sensitive to the impact of multimodal diversity involving geographical, sex, macrosocial, and disease-based factors from diverse populations, despite the heterogeneity in data acquisition and processing. Utilizing an deep learning architecture of the brain’s high-order interactions^1^ across fMRI and EEG signals, combined with globally accessible and affordable data, our study paves the way for more inclusive tools to assess disparities and diversity in brain aging. These tools can be vital in identifying MCI, Alzheimer’s disease and bvFTD risk factors, as well as to characterizing and staging disease processes. In the future, personalized medicine approaches could leverage models of BAGs to establish worldwide protocols for aging and neurocognitive disorders.

## Methods

The total dataset consisted of 5306 participants, with 2953 undergoing fMRI and 2353 EEG acquisitions. Of these, 3509 were controls, 517 had MCI, 828 Alzheimer’s disease, and 463 bvFTD.

### fMRI dataset

The fMRI study involved 2953 participants from both non-LAC (USA, China, Japan) and LAC (Argentina, Chile, Colombia, Mexico, Peru), including 1444 healthy controls (HC). Two hundred fifteen participants met the Petersen criteria for MCI with a 24 MMSE cut-off value, 719 were diagnosed as probable AD^61^, and 402 fulfilled the diagnostic criteria for bvFTD^62^. LAC participants were recruited from the Multi-Partner Consortium to Expand Dementia Research in Latin America (ReDLat, with participants from Mexico, Colombia, Peru, Chile, and Argentina)^63^. Non-LAC participants were non-Latino individuals from ReDLat, the Alzheimer’s Disease Neuroimaging Initiative (ADNI), and the Neuroimaging in Frontotemporal Dementia (NIFD) repository. The datasets were matched on sex, age, and years of education (Table 1). Sex information was determined by self-report. No information regarding gender was inquired. To ensure data reliability, we excluded subjects who reported a history of alcohol/drug abuse or psychiatric or other neurological illnesses. No participants reported a history of alcohol/drug abuse, psychiatric, or other neurological illnesses.

### EEG dataset

The total dataset involved 2353 participants. Controls comprised 1183 participants, including 737 from non-LAC (Turkey, Greece, Italy, United Kingdom, and Ireland) and 446 from LAC (Cuba, Colombia, Brazil, Argentina, and Chile). The participants presenting with clinical conditions were recruited from a multisite study with harmonized assessments^25,36,63^ in LAC (Argentina, Brazil, Chile, and Colombia). This dataset included 133 patients with MCI, 108 with Alzheimer’s disease, and 57 with bvFTD. The controls datasets were matched on age, sex, and years of education concerning the clinical groups (MCI, Alzheimer’s disease, and bvFTD) (Table 1). Sex information was determined by self-report. No information regarding gender was inquired. The Petersen criteria defined the MCI group with a 24 MMSE cut-off value. All individuals with Alzheimer’s disease met the criteria for probable disease following international diagnostic guidelines^61^. The bvFTD group met the diagnostic criteria for probable bvFTD^62^. No subject in any of the clinical conditions reported a history of alcohol/drug abuse, psychiatric, or other neurological illnesses.

### Ethics approval

The local institutions that contributed EEGs and/or fMRIs to this study approved the acquisitions and protocols (Supplementary Data S1), and all participants signed a consent form following the declaration of Helsinki. The overall study was approved by the consortium under multiple IRBs (FWA00028264, FWA00001035, FWA00028864, FWA00001113, FWA00010121, FWAA00014416, FWA00008475, FWA00029236, FWA00029089, and FWA00000068). Data collection and analysis posed no risks concerning stigmatization, incrimination, discrimination, animal welfare, environmental, health, safety, security, or personal concerns. No transfer of biological materials, cultural artifacts, or traditional knowledge occurred. The authors reviewed pertinent studies from all countries while preparing the manuscript.

### fMRI preprocessing

The images were obtained from different scanners and in distinct acquisition settings (Supplementary Table 4). We included two resting-state recordings, closed and open eyes, to increase the sample size for rs-fMRI data. The type of resting-state recording was controlled by a dummy variable (open or closed eyes) when employing the functional connectivity metric^64^. The resting state of fMRI preprocessing was conducted using the *fmriprep* toolbox (version 22.0.2). Furthermore, additional preprocessing was performed using the toolbox CONN22^64^. The CONN toolbox preprocessing included smoothing with a Gaussian kernel of 6 × 6 × 6 mm, the signal denoising through linear regression to account for confounding effects of white matter, cerebrospinal fluid, realignment, and scrubbing. A band-pass filter (0.008–0.09) Hz was also applied. After time-series preprocessing, we employed region-of-interest (ROI) analysis based on the brain regions of the Automated Anatomical Labeling (AAL90) atlas to reduce the dimensionality of the fMRI data for machine learning algorithms.

### EEG preprocessing

EEGs were processed offline using procedures implemented in a custom, automatic pipeline for computing brain functional connectivity in the EEG using a mesh model for multiple electrode arrays and source space estimation (see Supplementary Table 5 for acquisition parameters). The pipeline allows for the multicentric assessment of rsEEG connectivity and has been validated in a large-scale evaluation of connectivity in dementia^65^. Recordings were re-referenced to the average reference and band-pass filtered between 0.5 and 40 Hz using a zero-phase shift Butterworth filter of order 8. Data were downsampled to 512 Hz, referenced using the reference electrode standardization technique (REST), and corrected for cardiac, ocular, and muscular artifacts using two methods based on Independent Component Analysis (ICA). ICLabel (a tool for classifying EEG independent components into signals and different noise categories)^66^, and EyeCatch (a tool for identifying eye-related ICA scalp maps) were used^67^. Data were visually inspected after artifact correction, and malfunctioning channels were identified and replaced using weighted spherical interpolations.

EEG normalization: Following guidelines for multicentric studies^37^, EEG was rescaled to reduce cross-site variability. The normalization was carried out separately for each dataset and consisted of the Z-score transformation of the EEG time series. The Z-score describes the position of raw data in terms of its distance from the mean when measured in standard deviation units. The Z-score transformed EEG connectivity matrices display more prominent interhemispheric asymmetry and reinforced long-distance connections than unweighted connectivity representations^65^.

EEG source space estimation: The source analysis of the rsEEG was conducted using the standardized Low-Resolution Electromagnetic Tomography method (sLORETA). sLORETA allows estimating the standardized current density at each of the predefined virtual sensors located in the cortical gray matter and the hippocampus of a reference brain (MNI 305, Brain Imaging Centre, Montreal Neurologic Institute) based on the linear, weighted sum of a particular scalp voltage distribution or the EEG cross-spectrum at the sensor level. sLORETA is a distributed EEG inverse solution method based on an appropriate standardized version of the minimum norm current density estimation. sLORETA overcomes problems intrinsic to the estimation of deep sources of EEG and provides exact localization to test seeds, albeit with a high correlation between neighboring generators.

The different electrode layouts were registered onto the scalp MNI152 coordinates. A signal-to-noise ratio of 1 was chosen for the regularization method used to compute the sLORETA transformation matrix (forward operator for the inverse solution problem). The standardized current density maps were obtained using a head model of three concentric spheres in a predefined source space of 6242 voxels (voxel size = 5mm^3^) of the MNI average brain. A brain segmentation of 82 anatomic compartments (subcortical and cortical areas) was implemented using the automated anatomical labeling (AAL90) atlas. Current densities were estimated for the 153600 voltage distributions comprising the five minutes of rsEEG (sampled at 512 Hz). The voxels belonging to the same AAL region were averaged such that a single (mean) time series was obtained for each cortical region^32,68,69^.

### High-order interactions

After preprocessing 82 time-series from the AAL brain parcellation for each modality (fMRI and EEG), we calculated the high-order interactions across triplets composed of a region *i* and region *j* and a set comprising all the brain regions without *i* and *j*. To this end, we evaluated high-order interactions using the organizational information (*Ω*) metric. It is a multivariate extension of Shannon’s mutual information, which assesses the dominant characteristic of multivariate systems (i.e., high-order interactions). In this case, to operationalize the Shannon Entropy, we used the Gaussian copula approximation, which estimates the differential Shannon’s entropy from the covariance matrix of the Gaussian copula transformed data^70^. This is a mixture of a parametric and a non-parametric approach, as the copula is preserved in a non-parametric way but is then used to generate Gaussian marginals. The Ω quantifies the balance between redundancy and synergy in high-order interactions among brain regions. By definition, Ω > 0 implies that the interdependencies are better described as shared randomness, indicating redundancy dominance. Conversely, Ω < 0 suggests that the interdependencies are better explained as collective constraints, indicating synergy dominance. After normalization, its magnitude ranges from −1 to 1. The Ω can be expressed as:

(1) $$\Omega \left(X^{n}\right)=(n-2)H\left(X^{n}\right)+\sum_{j=1}^{n}\left[H\left(X_{j}\right)-H\left(X_{-j}^{n}\right)\right],$$

where *X*^*n*^ is the random vector that describes the system, and *H* is the Shannon’s entropy. When *n* is reduced to three variables (*x*, *y*, and *z*), *Ω* can be expressed as

(2) Ω(x,y,z)=H(x,y,z)−H(x,y)−H(x,z)−H(y,z)+H(x)+H(y)+H(z).

To analyze brain activity, *z* can be considered a multivariate time series representing the activity of all brain regions except for *x* and *y*. Therefore, *O info* measures how synergistic or redundant is the relationship between two brain regions concerning the rest of the regions.

### Model input preprocessing

As input to the models, the weighted adjacency matrix corresponding to the Ω metric was converted to a graph. This matrix defines the edges in the graph, where the weight of each edge reflects the Ω value between the corresponding regions. The feature vectors at each graph node are derived from the O-info matrix; specifically, each node’s feature vector is the corresponding row in the Ω matrix. To this end, the connectivity matrices were first converted to tensors using the PyTorch deep learning library, enabling their efficient manipulation. Subsequently, these tensors were reshaped, organizing the connectivity data into a structure where each tensor represented the features of nodes within a graph. This transformation preserved the relational information from the original matrices, making it accessible for analysis by graph neural networks. To ensure the integrity of the data, graphs containing NaN values, either in their features or target values, were filtered out. The remaining graphs were then split into training and validation sets using a stratified split to ensure a balanced representation of age groups in both sets.

### Data augmentation

We employed augmentation tailored for connectivity matrices to make the model more resilient to heterogeneity and generalizability. Linear interpolation between matrices corresponding to neighboring age values was used, in contrast to traditional image augmentation techniques such as random rotations or crops that are inappropriate for connectivity data.

Given two matrices, *M*_*1*_ and *M*_*2*_, representing fMRI or EEG connectivity at ages *a*_*1*_ and *a*_*2*_, respectively, the interpolation to produce a matrix for a target age where *a*_*1*_ < *a*_*t*_ < *a*_*2*_ was conducted using the formula:

(3) $$M_{t}=(1-\alpha )M_{1}+\alpha M_{2}$$

Here, $\alpha =\frac{a_{t}-a_{1}}{a_{2}-a_{1}}$ represents the interpolation factor.

This augmentation method enabled the generation of fMRI and EEG connectivity matrices for age values previously absent in the data set. The derived matrices, through interpolation, ensure a smooth transition in the fMRI and EEG patterns from one age value to another, thereby maintaining the inherent physiological significance of the original data—preliminary validation against a hold-out dataset showed improvements in model fit against dataset heterogeneity. We included 500 samples with data augmentation only the training datasets for both modalities, half for the non-LAC and half for the LAC samples.

### The architecture of the models

Two Graph Convolutional Networks (GCNs)^71^ were designed for this study, specifically tailored to process graph-structured data. We employed the PyTorch Geometric code library based on the PyTorch library to develop and train the models. Two models were created, one for the fMRI data and another for the EEG data. Unlike traditional convolutional networks suited for neuroimaging data, functional connectivity demands a specialized approach since neighboring data points are not necessarily close in native space (i.e., adjacent brain areas). The GCN employs adjacency matrices of graphs as inputs comprised of node features. Each node in the graph aggregates features from its neighbors through a series of operations, including multiplication by a normalized adjacency matrix, transformation using a weight matrix, and applying an activation function, here the ReLU^72^. The architecture employed in our work consisted of two Graph Convolutional layers. The input features (O-info matrix) were passed through the first convolutional layer, followed by a ReLU activation function and a dropout layer for regularization. The features were then passed through the second convolutional layer. Finally, average pooling was used to aggregate the output features. To train the two models, we combined Mean Squared Error (MSE) as the loss function and the Adam optimizer. Given the variability in the data and potential model configurations, we implemented a hyperparameter tuning process using a grid search over specified learning rates and epoch numbers. For each model for the controls, the data was initially split into 80% for training and validation, and 20% for hold-out testing. Within the 80% training and validation set, we applied 5-fold cross-validation to determine the optimal hyperparameters for the model. After determining the best hyperparameters through this cross-validation process, the final model’s performance was evaluated on the remaining 20% hold-out test set to assess its generalization capability^73^.

### Statistical analyses

Following hyperparameter tuning, each model was retrained using the best hyperparameters on the training set and evaluated on the test set. For a more comprehensive assessment, the predicted age values were compared to the actual age values using Pearson’s correlation coefficient, R-squared, and Cohen’s *f*^*2*^ effect size for each model^74^. We used the method outlined below to evaluate if the model was predicting increased or decreased ages concerning the actual chronological age.

The Mean Directional Error (MDE) is a diagnostic metric used to evaluate the prediction accuracy of the models, specifically focusing on the direction of prediction gaps rather than their magnitude to detect bias. It is calculated as follows:

(4) $$MDE=\frac{1}{n}\sum_{i=1}^{n}\left(y_{i}-{\hat{y}}_{2}\right)$$

The function “sign” yields a value of +1 if the prediction is above the actual value, −1 if below, and 0 if they are equal. *y*_*i*_ is the real age of subject *i* and *ŷ*_*i*_ is the predicted age. An MDE value close to zero suggests a balanced number of overestimations and underestimations. Positive or negative values indicate systematic biases in the prediction method, where a positive MDE means the model generally overpredicts, and a negative MDE indicates underprediction.

In our analysis when comparing models, we sought to examine potential regional biases in predictive accuracy and compare possible sex effects or signal acquisition noise. The statistical approach involved conducting permutation tests (5,000 subsample iterations each), a non-parametric statistical test that does not assume a specific distribution of the data, thus offering flexibility in handling non-normal distributions. Given the nature of the permutation test, our analysis constituted two-sided tests, assessing the likelihood of observing the obtained difference under the null hypothesis of no difference between the models. While the permutation test alleviates the need for normality assumptions, making it resilient to deviations from normal distribution, it inherently addresses multiple comparison concerns by evaluating the empirical distribution of the test statistic under the null hypothesis.

We compared the adequacy of the models employing the root mean square error (RMSE). This is a metric to quantify the discrepancies between predicted and observed values in modeling, given by the formula:

(6) $$RMSE=\sqrt{\frac{1}{N}\sum_{i=1}^{n}{\left(y_{i}-{\hat{y}}_{i}\right)}^{2}}$$

In this equation, *y*_*i*_ is the observed value, *ŷ*_*i*_ is the predicted value, and *N* is the total number of observations. RMSE measures the average magnitude of errors between predicted and actual observations. The squaring process results in a higher weight to outliers, making it a useful measure to evaluate if a model is robust to outliers.

To evaluate feature importance, we employed bootstrapping to assess the significance of individual nodes (i.e., brain areas) and edges (i.e., connections between brain nodes/regions) within the graph neural network. With this approach, we executed a two-step process to quantify the node and its edge’s impact on the model’s predictions. Initially, the model’s output was calculated with all nodes and its edges present to establish a baseline performance metric. Subsequently, the analysis was repeated after removing each node and edge at a time, thus simulating network information absence. The difference in the model’s output, with and without each area and edge was quantified, providing a measure of the network node importance. This process was repeated across multiple bootstrap testing dataset samples (n=5000) to calculate confidence intervals. Finally, a feature importance list of nodes was generated in descending order of importance for brain age prediction. This methodological framework allowed for an analysis of network-level contributions to each model’s overall predictive performance.

#### Gradient boosting regression models

We used gradient boosting regression models^75^ to investigate the impact of factors associated with the physical and social exposomes, and disease disparities, on BAGs between LAC and non-LAC populations. As predictors, we included country-level measures of: (i) air pollution (PM2.5 exposure), (ii) socioeconomic inequality (the GINI index)^76^, (iii) the burden of communicable, maternal, prenatal, and nutritional conditions, and (iv) the burden of non-communicable diseases. These indicators were sourced from the updated country-specific data provided on the World Bank’s platform (https://databank.worldbank.org/). Additionally, individual neurocognitive status (being controls versus having Alzheimer’s disease, MCI, or bvFTD) was included as predictor. BAGs from fMRI and EEG datasets were the outcomes.

Models were trained using 90% of the dataset and subsequently tested on an independent 10% subset, employing a 10-fold cross-validation framework. The cross-validation was repeated 10 times. Within each iteration, estimation coefficients for the predictors, as well as the R-squared, Cohen’s f^277^, and RMSE, were computed. We assessed feature importance using a multi-method approach incorporating permutation importance, features importance based on the mean decrease in impurity (MDI), and SHAP values^78^. We provided the mean importance values for each method, along with their 99% confidence interval, as well as the average R-squared and Cohen’s f^277^. Features whose lower confidence interval boundary crosses zero are considered non-significant. In order to optimize Ridge’s hyperparameters, Bayesian optimization was employed. Following the same multi-method approach, we conducted gradient boosting regressions to explore the effect of gender inequality and sex on BAGs. As predictors, we included: (i) the country level gender inequality index (GII), a composite metric measuring reproductive health, empowerment and the labor market, (ii) sex, (iii) region (LAC vs non-LAC) and (iv) individual neurocognitive status (HC versus Alzheimer’s disease, MCI, or bvFTD). BAGs from fMRI and EEG were the outcomes

#### Data quality assessment

For the fMRI overall data quality (ODQ) metric, each timeseries was segmented in 20 repetition time (TR) length to evaluate the temporal signal-to-noise ratio (tSNR)^79^, which is calculated as the mean fMRI signal divided by its standard deviation within each segment. Segments with tSNR above a threshold of 50 were classified as high quality^79^. As additional evaluations to consider overall acquisition quality, we checked the variability of the tSNR segments of all the time series in the brain to check for spatial consistency. Lastly, we checked for remaining outliers as signal spikes from movement or transient gradient artifacts. Thus, the fMRI ODQ was computed as a percentage of good segments considering its tSNR, low spatial variability, and the number of segments not having spikes from movement or transient gradient remaining artifacts.

For the EEG data quality assessment, we followed the method proposed by Zhao et al^80^. The EEG signals were divided into 1-second segments, and the quality of each segment was evaluated using four specific metrics. These metrics included the detection of weak or constant signals based on standard deviation, the identification of artifacts through signal amplitude ratios, the presence of high-frequency noise, and low correlation between channels. The EEG ODQ was then calculated as the percentage of segments exhibiting good quality. A value of 0 indicated that all segments were of poor quality, while a value of 100 indicated that all segments were of high quality.

#### Sensitivity analyses

We examined whether variations in fMRI or EEG data quality explained the differences in brain age between the non-LAC and LAC, comparing different groups’ fMRI^79^ and EEG^80^ data quality metrics, with subsample permutation tests with 5000 iterations for each comparison. In addition, we conducted a linear regression to examine the association between the fMRI data quality metrics and the BAGs. To further control for scanner effects, we implemented an additional harmonization strategy in the fMRI training dataset. This method involves normalizing the BAG variable within each scanner type by scaling the data to a fixed range using the min-max scaler^14^. This ensures that the minimum and maximum values of the BAG variable are consistent across different scanners, thereby reducing variability due to scanner differences. Additionally, we accounted for the sign of the BAG after normalization to maintain the interpretability of positive and negative values. This procedure adjusts for location and scale differences (e.g., mean and variance) across sites, minimizing scanner-related variability.

We used permutation tests (5000 subsample iterations each) to compare the BAGs between subsamples of participants undergoing fMRI with open versus closed eyes. We included 124 controls with closed eyes and 86 with open eyes, 269 Alzheimer’s disease with closed eyes and 164 with open eyes, and 88 bvFTD with closed eyes and 69 with open eyes. Notably, all MCI participants underwent fMRI with open eyes. Our findings revealed no significant differences in BAGs when analyzing data from open versus closed eyes conditions across all group comparisons (permutation test = 5000 iterations).

### Ethics and inclusion statement

This work involved a collaboration between researchers in multiple countries. Contributors from different sites are included as coauthors according to their contributions. Researchers residing in LMIC were involved in study design, study implementation, methodological procedure, writing and reviewing processes. The current research is locally relevant due to the larger disparities observed in LAC. Roles and responsibilities were agreed among collaborators ahead of the research. Ethics committees approved all research involving participants. To prevent any stigmatization, all identifying information has been removed to preserve the privacy of individuals. We endorse the Nature Portfolio journals’ guidance on LMIC authorship and inclusion. Authorship was based on the intellectual contribution, commitment, and involvement of each researcher in this study. We included authors born in LMICs and other underrepresented countries.

## Extended Data

**Extended Data Fig. 1.** Associations of sex and gender inequality with BAGs. Multi-method approach comprising SHAP values, features and permutation importance. Plot shows the mean importance values for each method, along with their 99% confidence interval, as well as the average R-squared and Cohen’s F^2^. Having a neurocognitive disorder, being female, and living in countries with larger gender inequality (particularly from LAC), were associated with higher BAGs. LAC = Latin American countries.

**Extended Data Fig. 2.** Prediction gaps between fMRI datasets with either eyes open or eyes closed protocols. No significant differences were observed between participants with open vs. closed eyes within the same groups (permutation test = 5000 iterations). * p < 0.05, ** p < 0.01, *** p < 0.001. LAC = Latin American countries, OE = open eyes, CE = closed eyes.

**Extended Data Fig. 3.** BAGs between subsamples of mild cognitive impairment (MCI) and Alzheimer’s disease (AD) groups matched by chronological age. Results were similar to those reported for the total MCI and Alzheimer’s disease datasets in Figs. 4a and b (permutation test = 5000 iterations).

## Figures and Tables

**Table 1.** Demographics for fMRI and EEG datasets

Full dataset							
All participants n = 5306		HCs = 3509	MCI = 517	AD = 828	bvFTD = 463		
fMRI dataset							
Variable		HCs Non-LAC n = 967 LAC n = 477	MCI Non-LAC n = 215 LAC n = 169	AD Non-LAC n = 214 LAC n = 505	bvFTD Non-LAC n = 190 LAC n = 216	Statistics Non-LAC vs. LAC	Post-hoc comparisons
Sex (F:M)	Non-LAC	470:497	114:101	112:102	98:92	*χ*^2^ = 2.19	HC-MCI: *p* > 0.05
						*p* = 0.533	HC-AD: *p* > 0.05
							HC-bvFTD: *p* > 0.05
	LAC	261:216	84:85	262:243	105:111	*χ*^2^ = 2.76	HC-MCI: *p* > 0.05
						*p* = 0.429	HC-AD: *p* > 0.05
							HC-bvFTD: *p* > 0.05
Age (years)	Non-LAC	53.55	59.62	76.59	73.14	F = 3.13	HC-MCI: *p* > 0.05
		(13.43)	(8.77)	(9.35)	(8.56)	*p* = 0.47	HC-AD: *p* > 0.05
						*np*^*2*^ = 0.02	HC-bvFTD: *p* > 0.05
Range: [22–91]	LAC	65.34	66.53	77.52	73.15	F = 3.62	HC-MCI: *p* > 0.05
		(11.44)	(8.18)	(9.35)	(8.76)	*p* = 0.45	HC-AD: *p* > 0.05
						*np*^*2*^ = 0.02	HC-bvFTD: *p* > 0.05
Years of Education	Non-LAC	13.15	14.15	13.12	11.16	F = 2.19	HC-MCI: *p* > 0.05
		(5.41)	(3.41)	(5.34)	(3.56)	*p* = 0.49	HC-AD: *p* > 0.05
						*np*^*2*^ = 0.02	HC-bvFTD: *p* > 0.05
Range: [0 – 25]	LAC	12.11	11.52	8.89	7.89	F = 1.31	HC-MCI: *p* > 0.05
		(3.39)	(6.32)	(4.34)	(3.36)	*p* = 0.68	HC-AD: *p* > 0.05
						*np*^*2*^ = 0.01	HC-bvFTD: *p* > 0.05
EEG dataset							
		HCs Non-LAC n = 569 LAC n = 1486	MCI LAC n = 133	AD LAC n = 108	bvFTD LAC n = 57	Statistics Non-LAC vs. LAC	Post-hoc comparisons
Sex (F:M)	Non-LAC	470:99	-	-	-	*χ*^2^ = 64.62	-
						*p* < 0.001*	
	LAC	954:532	111:22	85:23	39:18	*χ*^2^ = 28.05	HC-MCI: *p* > 0.05
						*p* < 0.001*	HC-AD: *p* > 0.05
							HC-bvFTD: *p* > 0.05
Age (years)	Non-LAC	58.98	-	-	-	t = 4.21	-
		(12.03)				*p* = 0.07	
						*np*^*2*^ = 0.02	
Range: [21–92]	LAC	66.74	62.54	78.62	71.05	F = 7.62	HC-MCI: *p* > 0.05
		(13.94)	(9.98)	(8.34)	(9.34)	*p* < 0.001*	HC-AD: *p* > 0.05
						*np*^*2*^ = 0.07	HC-bvFTD: *p* > 0.05
Years of Education	Non-LAC	14.85 (4.91	-	-	-	t = 3.54	-
						*p* = 0.08	
						*np*^*2*^ = 0.01	
Range: [0 – 24]	LAC	13.92	8.12	10.75	14.38	F = 6.31	HC-MCI: *p* > 0.05
		(3.39)	(4.34)	(6.32)	(5.49)	*p* < 0.001*	HC-AD: *p* > 0.05
						*np*^*2*^ = 0.06	HC-bvFTD: *p* > 0.05

Results are presented as mean (SD). Asterisks (*) indicate an alpha level of *p* < 0.05. Demographic data comparing non-LAC and LAC groups were assessed using unpaired t-tests, while data for pathological groups were analyzed using ANOVAs followed by Tukey post-hoc pairwise comparisons, except for sex, which was analyzed using Pearson’s chi-squared (χ^2^) test. Effect sizes were calculated using partial eta squared (ηp^2^). Abbreviations: HC = healthy control, MCI = mild cognitive impairment, AD = Alzheimer’s disease, bvFTD = behavioral variant frontotemporal dementia.

## Data Availability

All preprocessed data are openly available at: https://osf.io/8zjf4/. The fMRI and EEG datasets comprise sources (a) currently publicly available for direct download after registration and access application, (b) available after contacting the researcher, or (c) accessible after IRB approval of formal data-sharing agreement in a process that can last up to 12 weeks. The fMRI sources that are publicly available for direct download are the following: Alzheimer’s Disease Neuroimaging Initiative (ADNI) (USA) (ida.loni.usc.edu/collaboration/access/appLicense.jsp), Chinese Human Connectome Project (CHCP) (China) (scidb.cn/en/detail?dataSetId=f512d085f3d3452a9b14689e9997ca94#p2), The frontotemporal lobar degeneration neuroimaging initiative (FTLDNI) (USA) (ida.loni.usc.edu/collaboration/access/appLicense.jsp), and Japanese Strategic Research Program for the Promotion of Brain Science (SRPBS) (Japan) (bicr-resource.atr.jp/srpbsopen/). The fMRI sources available after contacting the researcher include ReDLat USA by contacting Bruce Miller at UCSF through datasharing@ucsf.edu. The fMRI sources that require IRB approval and a formal data sharing agreement include: ReDLat pros (Argentina, Chile, Colombia, Mexico, Peru) by contacting Agustín Ibañez at agustin.ibanez@gbhi.org, Centro de Gerociencia Salud Mental y Metabolismo (GERO) (Chile) by contacting Andrea Slachevsky at andrea.slachevsky@uchile.cl, ReDLat pre (Argentina) by contacting Agustín Ibañez at agustin.ibanez@gbhi.org, ReDLat pre (Peru) by contacting Nilton Custodio at ncustodio@ipn.pe, ReDLat pre (Colombia) by contacting Diana Matallana at dianamat@javeriana.edu.co, ReDLat pre (Colombia -II) by contacting Felipe Cardona at felipe.cardona@correounivalle.edu.co, ReDLat pre (Mexico) by contacting Ana Luisa Sosa at drasosa@hotmail.com, ReDLat pre (Chile) by contacting María Isabel Behrens at behrensl@uchile.cl, and ReDLat pre (Chile) by contacting Andrea Slachevsky at andrea.slachevsky@uchile.cl. The EEG sources that are publicly available for direct download are Centro de Neurociencias de Cuba (CHBMP) (Cuba) (www.synapse.org/Synapse:syn22324937). The EEG sources that are available after contacting the researcher include BrainLat (Argentina) by contacting Agustina Legaz at alegaz@udesa.edu.ar, BrainLat (Chile) by contacting Agustina Legaz at alegaz@udesa.edu.ar, Izmir University of Economics (Turkey) by contacting Gorsev Gener at gorsev.yener@ieu.edu.tr, Trinity College Dublin (Ireland) by contacting Francesca Farina at francesca.farina@northwestern.edu, Universidad de Antioquia (Colombia) by contacting Francisco Lopera at floperar@gmail.com, Universidad de Sao Paulo (Brazil) by contacting Mario Parra at mario.parra-rodriguez@strath.ac.uk, Universidad de Roma La Sapienza (Italy) by contacting Susana Lopez at susanna.lopez@uniroma1.it, University of Strathclyde (UK) by contacting Mario Parra at mario.parra-rodriguez@strath.ac.uk, Istanbul Medipol University (Turkey) by contacting Tuba Aktürk at takturk@medipol.edu.tr, and Takeda (Chile) by contacting Daniela Olivares at danielaolivaresvargas@gmail.com. For additional details, see **Supplementary Data S1**.
